# Building workforce capacity for the surgical management of cervical cancer in a fragile, low-income African nation—Democratic Republic of the Congo

**DOI:** 10.3332/ecancer.2021.1232

**Published:** 2021-05-13

**Authors:** Michael L Hicks, Alex Mutombo, Tankoy Gombo YouYou, Mukanya Mpalata Anaclet, Mulumba Kapuku Sylvain, Kabongo Mukuta Mathieu, Ronda Henry-Tillman, Dorothy Lombe, Maya M Hicks, Leeya Pinder, Louis Kanda, Mirielle Kanda, Groesbeck P Parham

**Affiliations:** 1 St Mary Mercy Cancer Center, 36475 Five Mile Rd, Livonia, MI 48154, USA; 2 Department of Obstetrics and Gynecology, University of North Carolina at Chapel Hill, 101 Manning Dr, Chapel Hill, NC 27514, USA; 3 Department of Obstetrics and Gynecology, University Teaching Hospital – Women and Newborn Hospital, 10101 Nationalist Way, Lusaka, Zambia; 4 St Joseph Mercy Oakland Cancer Center, 44405 Woodward Ave, Suite 202, Pontiac, MI 48324, USA; 5 McLaren Macomb Medical Center, 1000 Harrington Blvd, Mount Clemens, MI 48043, USA; 6 Biamba Marie Mutombo Hospital, No. 9777, Boulevard Lumumba, Commune de Masina, Kinshasa, Democratic Republic of the Congo; 7 Winthrop P Rockefeller Cancer Institute, University of Arkansas for Medical Sciences, 4301 West Markham St, Slot #725, Little Rock, AR 72205, USA; 8 Department of Oncology, University Teaching Hospital, Cancer Diseases Hospital, Lusaka Zambia PO Box Rw, 51337 Lusaka, Zambia; 9 Howard University College of Medicine, 520 W St NW, Washington, DC 20059, USA; 10 Department of Oncology, University of Washington, 1959 NE Pacific St, Seattle, WA 98195, USA; 11 Dikembe Mutombo Foundation, 400 Interstate N Pkwy, Suite 1040, Atlanta, GA 30339, USA; a https://orcid.org/0000-0002-1819-155X; b https://orcid.org/0000-0002-1782-9523; c https://orcid.org/0000-0002-5083-1801; d https://orcid.org/0000-0002-1993-3367; e https://orcid.org/0000-0001-5922-5990

**Keywords:** surgical capacity building, cervix cancer, LMIC cancer control in Africa, surgical training in LMIC, Democratic Republic of Congo, fragile, conflict and violence affected societies, African cancer center

## Abstract

**Objectives:**

Surgery is a cornerstone of the management of cervical cancer. Women diagnosed with cervical cancer in sub-Saharan Africa have very little access to specialised (gynaecologic oncology) surgical services. We describe our experiences and challenges of training local general gynaecologists to surgically treat early stage invasive cervical cancer at a private sector healthcare facility in a fragile, low-income African nation.

**Methods:**

Implementation of the training curriculum began with assigned self-directed learning. It continued with on-site training which consisted of preoperative surgical video reviews, pre- and intra-operative assessment of disease status, deconstruction of the designated surgical procedure into its critical subcomponents and trainees orally communicating the steps of the surgical procedure with the master trainers. High-volume repetition of a single surgical procedure over a short time interval, intra-operative bedside mentoring, post-operative case review and mental narration were critical to the process of surgical skills transfer.

**Results:**

Nineteen radical abdominal hysterectomies were successfully performed over four training visits; trainees were able to perform the procedure alone after eight cases; surgical complications decreased over time. The trainees have continued to perform the surgical procedures independently.

**Conclusion:**

Life-saving surgical capacity for the treatment of cervical cancer has been established and sustained at a private sector healthcare facility in a fragile, low-income African setting, through an innovative model of surgical training.

## Introduction

Worldwide, cervical cancer is a major public health problem, with over 500,000 new cases and 300,000 deaths occurring worldwide in 2018 [1]. One in every five of those deaths takes place in sub-Saharan Africa, the region with the world’s highest age-standardised cervical cancer incidence and mortality rates, and where the disease is the most commonly diagnosed malignancy and leading cause of cancer-related death in women [1]. In the African setting, cervical cancer occurs predominantly in women of reproductive age, many of whom are mothers of young children whose survivorship is negatively impacted by the premature deaths of their mothers [2]. The global cervical cancer burden is projected to continue to increase, rising to nearly 700,000 cases and 400,000 deaths in 2030, with analogous increases anticipated in future years. Most of the increase will occur in the world’s most impoverished regions [3]. To address this impending tragedy, the Director General of the World Health Organisation (WHO) issued a call for widespread implementation of proven and cost-effective measures to eliminate cervical cancer as a public health problem [4]. The WHO global strategy proposes the following *90-70-90* targets that need to be met by 2030 for countries to be on the path towards cervical cancer elimination:

- 90% of the girls fully vaccinated with the HPV vaccine by age 15;
- 70% of the women screened with a high-performance test by age 35, and again by age 45;
- 90% of the women identified with cervical disease receive treatment (90% of women with pre-cancer treated; 90% of women with invasive cancer managed).

Efforts are underway to initiate and scale programmes for the prevention and early detection of cervical cancer in African countries with the highest disease burdens. On the contrary, there has been little investment in building cancer management capacity, despite the present and increasing need for such. In a recent review of the present status of cancer treatment in Africa, the authors described it as ‘shocking and deplorable’ [5]. A prime example is the absence of any major investments by local African governments or international agencies in building surgical capacity and infrastructure for the treatment of early stage cervical cancer. In fact, it is estimated that approximately 93% of the population of sub-Saharan Africa lack access to safe, affordable and timely surgical care of even the most basic types [6]. Data from sub-Saharan African countries that have scaled cervical cancer screening services reveal that 2%–4% of those screened will have invasive cervical cancer, of which 40%–50% will be early stage and potentially curable with surgery alone [7]. However, due to severely limited investments in surgical oncology infrastructure, women residing in this region are unable to access safe, effective and timely cancer surgery [8, 9]. The need for innovative models that lead to the rapid creation of sustainable surgical oncology capacity, and the requisite supporting infrastructure, is paramount.

## Background

In January 2016, a joint venture was formed between the Dikembe Mutombo Foundation (DMF) and the Friends of Africa Inc. (FOA). DMF is a non-profit organisation located in Atlanta, GA, and is dedicated to improving the health, education and quality of life for the people of the Democratic Republic of the Congo (DRC) through primary healthcare and disease prevention, the promotion of health policy, health research and increased access to healthcare education. FOA is a US 501c3 non-profit organisation whose mission is to increase gynaecologic cancer prevention and treatment capacity in Africa and the African Diaspora besides innovative on-site education and clinical demonstrations. The goal of the joint venture was to build women’s cancer (cervical and breast) early detection and treatment capacity at the Biamba Marie Mutombo Hospital (BMMH) in Kinshasa, DRC. The hospital, opened in 2007, is the brain trust and vision of the National Basketball Association legend and Hall of Famer Dikembe Mutombo, a Congolese national, and his close advisors Drs. Louis and Mireille Kanda, with the assistance of his foundation staff Susan M. Johnson and Alicia V. Smith. As an initial step, DMF sponsored a scientific conference on the state of cervical cancer in the DRC, held in Kinshasa in 2016. Local and international stakeholders from industry, government and civil society were invited and made a presentation. Immediately afterwards, an assessment was made of the available human and clinical resources at the BMMH, as well as the conditions of the physical plant. Informed by the conference presentations, the outcomes of the hospital site visit, and consultations with the Ministry of Health, a strategy was developed to train local cadre (professional hospital staff) to organise and perform the following services: cervical cancer screening using visual inspection with acetic acid, treatment of cervical cancer precursors with thermal ablation or large loop excision of the transformation zone, punch biopsy of cervical lesions suspicious for cancer, staging and surgical treatment of early stage invasive cervical cancer and palliation of advanced stage disease. Secondary to major funding from the Howard G. Buffet Foundation, the scope of training was vastly expanded to include breast cancer early detection and treatment services, chemotherapy services, clinic infrastructure development, international educational opportunities for Congolese trainees and breast and cervical cancer early detection outreach activities.

This particular manuscript describes our experiences and the challenges we encountered in building surgical oncology capacity by training staff gynaecologists to perform radical abdominal hysterectomy and pelvic lymphadenectomy for early stage invasive cervical cancer through a novel, competency-based, intensive training curriculum designed to rapidly facilitate surgical skills transfer.

## Materials and methods

### Teaching model

We implemented the approach to rapid surgical training we previously used in Malawi [10].

**Figure 1.**
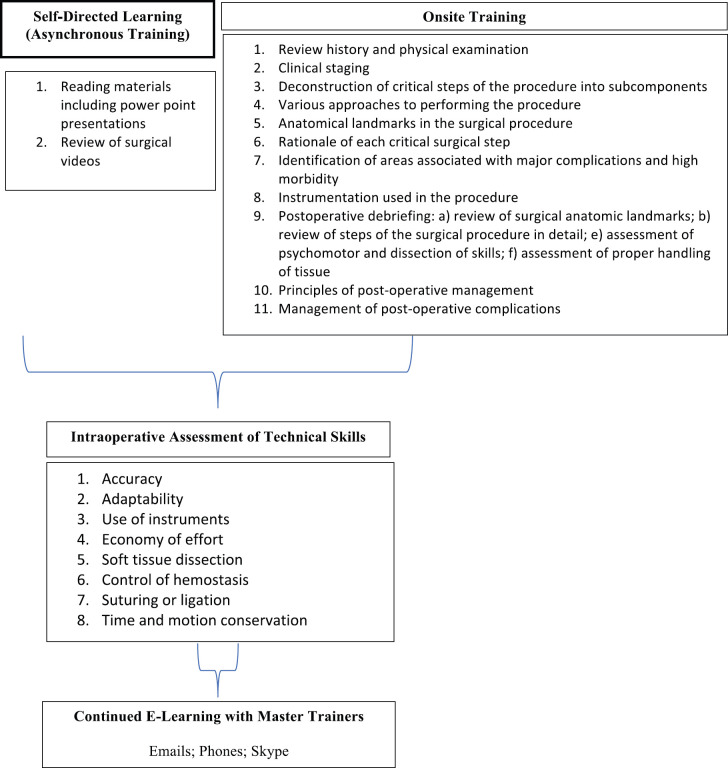
Overview of the competency-based surgical oncology training.

The core of the model is that experienced gynaecologic oncology master trainers train general gynaecologists to perform a single surgical procedure—radical abdominal hysterectomy, bilateral pelvic lymphadenectomy—using high-volume repetition of the procedure (several cases/day), over a short time interval. The DRC is a French-speaking nation which necessitated the translation of all written educational material into French. Additionally, a physician versed in English, French and gynaecology was hired as a language translator. Independent readings and video sessions were assigned 2 weeks before the arrival of the master trainers. The surgical procedure was reduced intellectually and schematically to its most critical and difficult constituent parts for clarity, then taught through active intra-operative mentoring in the surgical theatre (Figure 1 and Table 1). Each teaching case consisted of a trainee (general gynaecologist) performing the procedure under the guidance of a gynaecologic oncology master trainer, with relevant questioning and explanation of the rationale behind each step performed during the procedure. Throughout the training period, when not in surgery, the trainee was asked to perform, on their own, a mental narrative of the surgical procedure, several times a day, reviewing the rationale behind each step and mentally visualising potential intra-operative complications and corrective responses. Post-surgical debriefing of the operation was conducted after each case. Based on the immediate preoperative, intra-operative and post-operative assessment of the trainee, the master trainer determined the level of proficiency of the trainee related to performing the radical hysterectomy with pelvic lymphadenectomy, as a measure of determining when the trainee had reached a level of competency to perform the operation first alone but with close intra-operative observation, then alone without observation.

As an approach to teaching leadership, we stressed the importance of operating room control which consisted of establishing a code of ethics in the operative theatre, awareness of all pertinent activities and the elimination of any potential distractions. Additionally, we emphasised the significance of developing a team for accountable post-operative care of gynaecologic oncology patients. On-site clinical activity was followed by clinical mentorship through electronic communication, and subsequent quarterly follow-up visits by the master trainers.

### Training site

The surgical training programme was executed in a private sector hospital—BMMH—in the Democratic Republic of Congo, a 150-bed modern facility located in the capital city of Kinshasa. It serves a population of 6 million people in the immediate area, 17 million in the entire city of Kinshasa, as well as thousands more that travel from other provinces inside the DRC. Built by DMF, BMMH is a general-purpose hospital providing medical and surgical, inpatient, outpatient and emergency services for adults and children. It has treated over 500,000 patients for basic medical problems and emergencies. Cancer screening, diagnostics and treatments were non-existent prior to the implementation of our training programme.

A cervical cancer ‘screen and treat’ service platform was initially established in the facility, a few months prior to the launch of the surgical training curriculum, as previously described [7]. The established screening clinic was the source of all early stage cervical cancer cases referred to our programme for radical surgery or other forms of management.

### Personnel

The surgical trainees were Congolese board-certified obstetrician/gynaecologists who expressed a keen interest in learning how to perform a radical abdominal hysterectomy and pelvic lymphadenectomy. All were selected by the administration and governing board of BMMH. The purpose of the training was to transfer expertise of the designated surgical procedure to the identified staff gynaecologists, in order to build capacity and sustainability for the surgical treatment of early stage cervical cancer. The master trainers were two US board-certified gynaecologic oncologists (Michael Hicks and Groesbeck Parham) with over 50 years of surgical experience between them, much of which has been spent training and practicing medicine and teaching in US locations with high cervical cancer rates (Detroit, Michigan; South Central Los Angeles, California; Little Rock, Arkansas; and Birmingham, Alabama). Over the past 20 years, they have worked together in sub-Saharan Africa and the Caribbean islands, leading cervical cancer screening and surgical oncology demonstration seminars. Both were trained during a period when open laparotomy was the standard approach for the treatment of early stage cervical cancer. One (GP) has lived and worked as a gynaecologic oncologist in Zambia since 2005. Both are professors of gynaecologic oncology at the University of Zambia School of Medicine, where they have established an international training programme in gynaecologic oncology. They previously implemented this novel approach to rapid surgical skills transfer in Zambia and Malawi [10].

### Pre-operative assessment and post-operative care

All patients taken to surgery had a biopsy-proven tissue confirmation of cancer and underwent clinical staging consisting of abdominal pelvic examination, chest X-ray, pelvic ultrasound and blood chemistries. Those with stage IA2–IIA2 disease were medically evaluated, cleared for surgery, consented and then scheduled for radical abdominal hysterectomy, and bilateral pelvic lymphadenectomy. General anaesthesia and post-operative recovery were provided by staff physician-anaesthesiologists along with nurse anaesthetists. Post-operative care was provided by one of the selected surgical trainees along with a designated team.

## Results

Between 2017 and 2019, four site visits were made by the training team. Initial plans were to visit the facility for 1 week, every 3 months × 4. The itinerary had to be adjusted because of unpredictable outbreaks of political unrest and Ebola. Over the course of the four visits, 32 patients fulfilled the criteria for surgery. Their stages ranged between Ib1 and IIA2. Of the 32 patients who qualified, 19 underwent the scheduled procedure and the remaining 13 were no-shows and lost to follow-up. Pathology reports were available on 16/19 patients who underwent surgery. The histologic subtypes consisted of 12 squamous cell carcinomas and 4 adenocarcinomas. Pelvic lymph nodes were positive for metastatic disease in 7 (44%) patients—4/12 squamous cell carcinomas and 3/4 adenocarcinomas. The remaining nine (56%) had negative lymph nodes. Due to the absence of radiation therapy in the country, the seven patients with positive lymph nodes received cisplatin chemotherapy post-operatively. Surgical complications occurred in nine patients. Four had post-op wound infections, three ureteral injuries requiring re-implantation and two vesico-vaginal fistulae that required surgical repair. The range of blood loss was 450–1,000 ml. The trainees were able to perform the radical hysterectomy, pelvic lymphadenectomy with minimal supervision after performing the first eight cases with the master trainers. To date, they have continued performing surgeries independently of the master trainers.

## Discussion

The primary objective of our initiative was to build surgical oncologic capacity through the implementation and further validation of a training approach designed for rapid surgical skills transfer [10]. Using a non-traditional approach to surgical training that takes advantage of high volumes of disease, we accelerated the usual time required for a trainee to master the skills necessary to perform potential curative cervical cancer surgery. The result was the production of local surgeons capable of successfully performing the surgical intervention and the immediate establishment of a new surgical oncology service that was previously non-existent. The trainees were able to perform the operation by themselves after the second of four visits. We observed a slower time of transfer of knowledge in the DRC than in Malawi. We attribute this to the fact that during our immediate surgical interaction in the operating theatre, there was difficulty in understanding surgical instructions because of the language barrier, despite the presence of the interpreter. This improved as the master trainers began to better understand the local languages and gestures. We feel this is a very important factor to report because language and ethnography can influence the process of training candidates in non-English-speaking countries.

Of note, we were only able to perform 19 surgical procedures. Grant support was provided for training, travel and lodging of trainers, purchase of surgical and clinic equipment and supplies, subsidisation of selected hospital costs and remodelling clinic space. Despite the deep level of support, many patients were unable to afford the costs associated with travel or lodging in preparation for the surgery. Therefore, for pure economic reasons, many patients are unable to access life-saving surgery, even when the expertise is available. This is a major economic barrier for which there must be a solution, especially in low- and middle-income countries. Another barrier to care was the cost of pathology. Pathology infrastructure is presently being established at the hospital but at the time of the surgical training, specimens had to be sent to a private sector facility in Kinshasa.

Surgical complications were dominated by wound infections. Not all patients could afford prophylactic antibiotics, which potentially contributed to the high rate of wound infections. The ureteral injuries and vesico-vaginal fistulae were related to the inexperience of the trainees; however, these complications vanished as the trainees began to better understand pelvic anatomy, steps to the surgical procedure and the introduction of smaller surgical needles and dissecting instruments (e.g. scissors). Although it is never the objective to create complications, surgical repairs of these complications enhanced the depth and breadth of surgical training.

Working in a private sector medical facility allowed us to freely schedule training sessions without government restrictions. This improved the efficiency of the workflow. We were also able to explore opportunities to begin evaluating the use of low cost virtual telementoring. We experienced major impediments with camera optics for on-demand, real-time focusing in the operative theatre and inadequate internet band width to transmit images. Despite these limitations, we feel this is an area that can be explored in the future in order to assist in building surgical capacity from a distance.

## Conclusion

The DRC is the largest country in sub-Saharan Africa. Its people have faced some of the deadliest humanitarian crises in the world and it remains one of the poorest countries on the planet. In 2018, 72% of its population earned less than $2.00/day [11]. Despite the fragile nature of the economy, the dire poverty of its people, the Ebola crisis and political unrest as a backdrop, we were able to train local gynaecologists to perform a surgical procedure that is critical to the care of women with early stage invasive cervical cancer. By implementing a novel, intense surgical oncology training curriculum for Congolese healthcare professionals at BMMH, we contributed to the creation of a larger women’s oncology service (cervical cancer screening, breast early detection and surgical treatment, chemotherapy and palliative care) that is independently operational, financially sustainable and can potentially serve as a catalytic agent for future changes in cancer care in the DRC, such as the establishment of radiation therapy services. Using a matrix of on-site training, wise infrastructure investments and continued e-learning, a contextually appropriate training algorithm for the surgical treatment of early stage cervical cancer was designed and successfully implemented, providing accessibility to a critical life-saving service for women in the DRC.

## Conflicts of interest

None of the authors declare any conflicts of interest.

## Funding statement

Funding for the initiative was provided by a generous grant from the Howard G. Buffett Foundation, whose mission is to catalyze transformational change to improve the standard of living and quality of life, particularly for the world’s most impoverished and marginalized populations.

## Figures and Tables

**Table 1.** Subcomponents of the surgical procedure.

Mobilisation of the ureters from the medial leaves of the broad ligament peritoneum and parametrium to their points of entry into the bladder, including identification and ligation of the uterine arteries at their origin from the internal iliac arteries
Division of the cardinal ligaments and parametrium
Mobilisation of the rectum from the posterior cervix and vagina; identification and division of the uterosacral ligaments
Mobilisation of the bladder from the anterior cervix and vagina; identification and division of the vesico-uterine and vesico-cervical ligaments
